# An open experimental database for exploring inorganic materials

**DOI:** 10.1038/sdata.2018.53

**Published:** 2018-04-03

**Authors:** Andriy Zakutayev, Nick Wunder, Marcus Schwarting, John D. Perkins, Robert White, Kristin Munch, William Tumas, Caleb Phillips

**Affiliations:** 1 Materials Science Center, National Renewable Energy Laboratory, Golden, CO 80401, USA; 2 Computational Sciences Center, National Renewable Energy Laboratory, Golden, CO 80401, USA

**Keywords:** Semiconductors, Solar cells, Materials chemistry, Applied physics, Electronic devices

## Abstract

The use of advanced machine learning algorithms in experimental materials science is limited by the lack of sufficiently large and diverse datasets amenable to data mining. If publicly open, such data resources would also enable materials research by scientists without access to expensive experimental equipment. Here, we report on our progress towards a publicly open High Throughput Experimental Materials (HTEM) Database (htem.nrel.gov). This database currently contains 140,000 sample entries, characterized by structural (100,000), synthetic (80,000), chemical (70,000), and optoelectronic (50,000) properties of inorganic thin film materials, grouped in >4,000 sample entries across >100 materials systems; more than a half of these data are publicly available. This article shows how the HTEM database may enable scientists to explore materials by browsing web-based user interface and an application programming interface. This paper also describes a HTE approach to generating materials data, and discusses the laboratory information management system (LIMS), that underpin HTEM database. Finally, this manuscript illustrates how advanced machine learning algorithms can be adopted to materials science problems using this open data resource.

## Introduction

Machine learning is a branch of computer science concerned with algorithms that can develop models from the available data, reveal trends and correlation in this data, and make predictions about unavailable data. The predictions rely on data mining, the process of discovering patterns in large data sets using statistical methods. Machine learning methods have been recently successful in process automation, natural language processing, and computer vision, where large databases are available to support data-driven modeling efforts. These successes also sparked discussions about the potential of ‘Artificial Intelligence’ in science^1^ and ‘The Fourth Paradigm’^2^ of data-driven scientific discovery. In materials science, applying artificial intelligence to data-driven materials discovery is important, because new materials often underpin major advances in modern technologies. For example in advanced energy technologies, efficient solid state lighting was enabled by the use of gallium nitride in light-emitting diodes, electric cars were brought to life by intercalation materials used in lithium-ion batteries, and modern computers would not have been possible without the silicon material.

**Figure 1:**
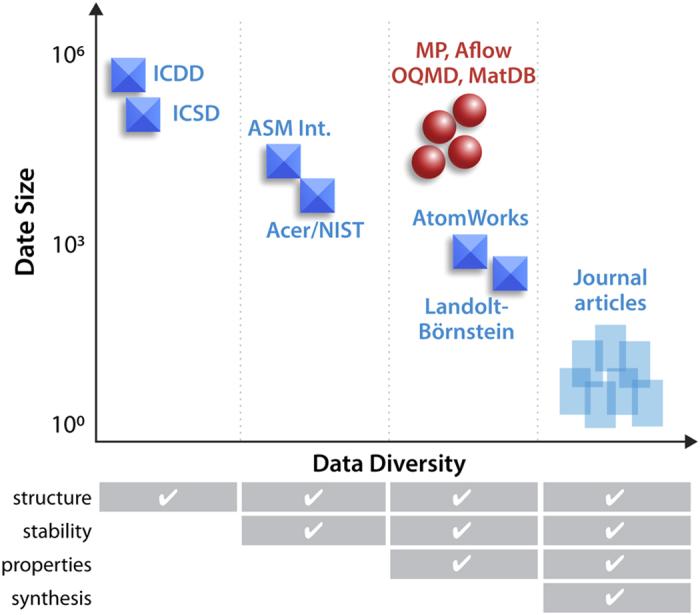
Schematic scatter plot of the size vs. diversity of materials data in existing databases. The computational databases (red circles) are large and diverse. In contrast, experimental databases are either large or diverse, limiting application of machine learning algorithms.

In computational materials science^3^, machine learning methods have been recently used to predict structure^4^, stability^5^, and properties^6^ of inorganic solid state materials. These results had been enabled by advances in simulation tools at multiple length scales^7^. The resulting simulated materials data are stored in ever-growing publically-accessible computational property databases^8–10^. In contrast to computations, experimental materials discovery using machine learning is limited by the dearth of large *and* diverse datasets (Fig. 1). Large experimental datasets like the Inorganic Crystal Structure Database (ICSD)^11^ contain 100,000's of entries, but are not diverse enough, as they contain only composition and structure of the materials. The diverse datasets like Landolt–Börnstein (http://materials.springer.com/)^12^ or AtomWorks (http://crystdb.nims.go.jp/index_en.html)^13^ contain 100's to 1000's of entries for different properties, so they are not large enough for training modern machine learning algorithms. Furthermore, none of these datasets contains synthesis information such as temperature or pressure, which is critical to making materials with target properties. Thus, machine learning for experimental materials research so far has focused on adoption of existing algorithms suitable for relatively small but complex datasets, such as collections of x-ray diffraction patterns^14^, microscopy images^15^, or materials microstructure^16^.

One potential machine learning solution to create large *and* diverse materials datasets is natural language processing^17,18^ from research articles published in scientific literature. However, the overwhelming majority of journal publications in experimental materials science is limited to what authors subjectively perceive as the most interesting of results, leading to large amounts of unpublished ‘dark data’^19^. Furthermore, the published papers are often biased towards positive research results^20^, since the publication of ‘failed’ experiments is discouraged in scientific literature. Such biased exclusion of negative results is a problem for machine learning, because many algorithms require both positive and negative results for efficient training. Finally, very small fraction of these journal article publications are linked to the corresponding publications of underlying data, despite the increasing requirements from funding agencies^21^ and encouragement from scientific journals^22^ to make research data available to the public.

Here we describe our progress towards the High Throughput Experimental Materials (HTEM) Database (htem.nrel.gov). The HTEM DB is the first publicly available large collection of experimental data for inorganic materials synthesized using high-throughput experimental (HTE) thin film techniques. Currently, HTEM DB contains information about synthesis conditions, chemical composition, crystal structure and optoelectronic property measurements of the materials. First, we provide an overview of the HTEM DB content, generated using the HTE experiments, and collected using materials data infrastructure. Next, we explain how to find, filter, and visualize the database content using its web interface on the example of one dataset. Finally, we illustrate how to perform machine learning on these data, using both supervised and unsupervised algorithms. More detailed technical information about the underlying experimental and data infrastructure of HTEM DB, as well as the machine learning algorithms is provided in the Methods section of the paper.

## Results

### Database Infrastructure and Content

**Figure 2:**
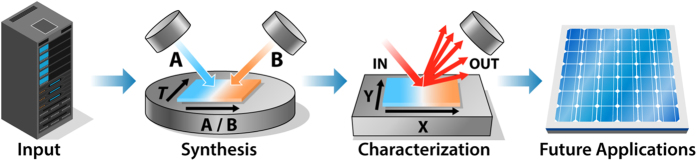
Schematic illustration of high-throughput synthesis and characterization thin film approach, with composition and temperature gradients across the substrate. The materials for the HTE experiments are selected based on inputs from computations or literature, and the outputs of the HTE experimentation are the candidate materials for further optimization for specific applications.

HTEM DB contains information about inorganic materials synthesized in thin film form, a combination that lends itself to high-throughput experimentation. The thin film sample libraries included in HTEM DB (semiconductors, metals and insulators) are synthesized using combinatorial physical vapor deposition (PVD) methods, and each individual sample on the library is measured using spatially-resolved characterization techniques (Fig. 2). The selection of the materials to be synthesized is usually based on computational predictions or prior experimental literature, and on specific target applications (e.g., solar cell materials, transparent contacts, piezoelectrics, photoelectrochemical absorbers etc.). Promising materials that result from the combinatorial synthesis and spatially resolved characterization are often optimized further using more traditional experimentation methods. A brief description of these high-throughput experimental techniques is provided in the Method section and in specialized review articles^23^, with the focus on electronic and energy materials. Other classes of materials that are amenable to high-throughput experimentation, such as organic polymers^24^ or homogeneous catalysts^25^, are not discussed in this paper because they are currently not included in HTEM DB.

**Figure 3:**
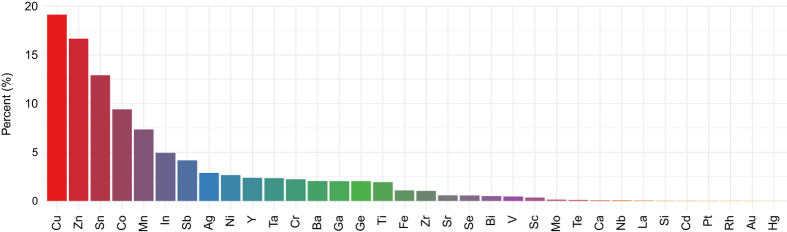
Metallic elements present in the HTEM DB, as determined from x-ray fluorescence measurement data and deposition precursor metadata. The majority of these elements are in form of oxide, nitride or chalcogenide compounds, with a few intermetallic compounds.

As of 2018, there are 141,574 entries of thin film inorganic materials in HTEM DB. These entries are arranged in 4,356 sample libraries, distributed across approximately 100 unique materials systems. The content of HTEM DB is graphically summarized in Fig. 3 in a form of a bar-chart of the 28 most common metallic elements. The majority of these metallic elements appear in the form of compounds (oxides 45%, chalcogenides 30%, nitrides 20%), but a few also form intermetallics (5%). The reported data for these materials include synthesis conditions such as temperature (83,600), x-ray diffraction patterns (100,848), composition and thickness (72,952), optical absorption spectra (55,352), and electrical conductivities (32,912). Out of all the sample entries, only about 10% have been described in peer-reviewed literature, and the remaining 80–90% have not been published before. Currently, more than a half (50–60%) of all the measured properties are publicly available; the rest belongs to privately-funded projects and to ongoing- or legacy public projects, for which data still needs to be curated.

**Figure 4:**
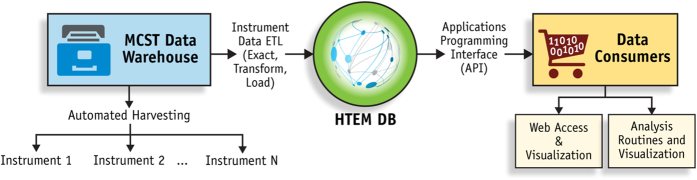
NREL Laboratory Information Management System (LIMS) for materials research enables the HTEM DB. The LIMS system is responsible for automatically harvesting, indexing and archiving measurement data and synthesis metadata into data warehouse. The extract-transform-load (ETL) process aggregates selected data in a custom relational database (HTEM DB). The HTEM DB is accessed by web-based user interface and other analysis and visualization programs via a standards-based API.

As shown in Fig. 4, HTEM DB described here leverages NREL’s custom laboratory information management system (LIMS), developed over many years in close collaboration between materials researchers, database architects, programmers, and computer scientists. First, the materials data is automatically harvested from synthesis and characterization instruments into a data warehouse—an archive of materials data and metadata files. Next, the extract-transform-load (ETL) process aligns synthesis and characterization data and metadata into the HTEM database with object-relational architecture. Finally, an application programming interface (API) is used for consistent interaction between client applications of data consumers (e.g., web user interface, statistical analysis programs) and the HTEM database. For example, the web user interface (web-UI) at htem.nrel.gov provides the interface for materials scientists around the world without access to unique high throughput experimentation equipment, to visualize a few selected datasets at a time, even if they did not generate these data. As another example, the API at hrem-api.nrel.gov allows computer scientists to access larger number of material datasets for data mining and machine learning purposes. Both of these types of access to large and diverse materials data in HTEM DB are likely to unleash creativity of researchers in unexpected ways that are difficult to foresee at the moment. More technical details about the database infrastructure and underlying information management system are provided in the Methods section.

### Database Exploration and Use

The most common mode of user interaction with HTEM DB is through its custom web-based interface at htem.nrel.gov. First, the database can be searched for the sample libraries of materials containing the elements of interest. Second, the search results can be filtered based on a number of criteria, including synthesis conditions, data quality/completeness, and other metadata. Finally, the filtered search results can be visualized and analyzed interactively, or downloaded for further more detailed analysis on the scientist’s computer. Below, we briefly discuss each of these web-interface features as of 2018; please refer to htem.nrel.gov for more updated information.

**Figure 5:**
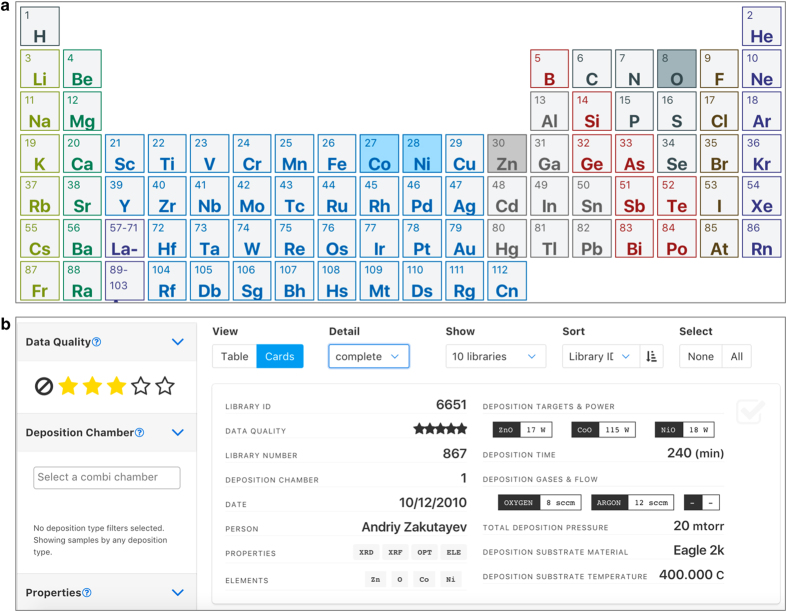
Examples of search and filter pages of the current version of HTEM DB. The search page (**a**) enables users to select the elements of interest, whereas the filter page (**b**) facilitates down-selection of the search results based on deposition conditions and other materials metadata.

The first step of interacting with the HTEM DB is to search for the sample libraries for the material of interest (Fig. 5a). The landing ‘Search’ page shows a periodic table where the elements can be selected with either ‘all’ or ‘any’ search option. The ‘all’ search option means that all the selected elements (and potentially other elements) have to be present in the sample in order to get search results. The ‘any’ search option means that as long as any of the selected elements are present, the search will return results. More advanced search logic is planned to be added in the future. At the top of the search page (as well as all subsequent pages), general information about HTEM DB can be found, including About, Stats, and API. This information is regularly updated, in contrast to this paper, so users are encouraged to check there frequently for any updates or changes of HTEM DB.

Once the search is performed, the resulting ‘Filter’ page (Fig. 5b) shows the list of sample libraries that meet the search criteria, and includes a sidebar for further down-selection of the results. The list of sample libraries has three possible views, each with progressively larger number of descriptors: compact (database sample ID, data quality, measured properties, included elements), detailed (+deposition chamber, sample number, synthesis/measurement date, and the person that generated the sample), and complete (+synthesis parameters such as targets/power, gasses/flows, substrate/temperature, pressure, and time). The five-star data quality scale (3-star value for uncurated data) was introduced in an attempt to enable each user to find their own balance between the quantity and the quality of the data being analyzed. This and all other descriptors can be used to sort the results of the search, or down-select them using the sidebar on the left hand side of the page. In addition, the sample library can be manually selected or unselected using the check mark boxes on the right hand side of the page. Once the filtering process is complete, the search results can be shared with other users using ‘Share Filter’ option that generates a unique web link, and the properties of the down-selected sample libraries can be explored by clicking the ‘Visualize’ button. Because of the large amount of available data, it is recommended to limit the number of the visualized sample libraries to less than 10–20 at this time, to avoid long loading times.

The properties view of the ‘Visualize’ page of the HTEM DB is a set of three user-definable plots for each selected sample library. For each of these three groups of plots, y-axis, x-axis, a color scale, and a point size can be defined using the drop-down menus at the ‘Visualize’ page. Both linear and logarithmic plots are available. Examples of the scalar variables that can be plotted in this way currently include x/y coordinates on the sample on the library, chemical composition of each constituent element, sample thickness, and its various properties, including electrical (sheet resistance, its standard deviation, resistivity, conductivity, etc), optical (e.g., band gap, average visible transmittance), and structural (number of peaks in XRD pattern). Multi-library plots for comparing multiple sample libraries on one plot are planned for the future. The spectra view of the Properties' page is arranged in a similar way as the properties view, with plots corresponding to structural information (i.e., x-ray diffraction patterns) and optical properties (i.e., absorption spectra) for each of the selected sample libraries, plottable on either linear or logarithmic scale. Currently, the axes of these plots cannot be defined by the user, however this feature is planned for the future.

**Figure 6:**
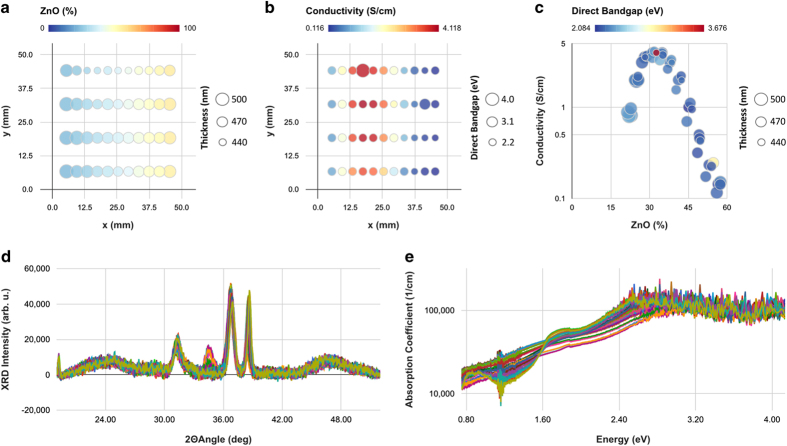
Example property and spectra plots in HTEM DB for one Zn-Ni-Co-O sample library. Panel (**a**) is a composition (color) and thickness (size), panel (**b**) is conductivity (color) and its direct bandgap (size), both as a function of x and y position on the sample library. Panel (**c**) is the summary analysis plot of logarithm of conductivity vs composition, with direct band gap as a color scale, and thickness as point size. Panels (**d**) and (**e**) are the underlying x-ray diffraction patterns, and the optical absorption spectra, respectively.

Clicking the ‘Library Details’ button on either ‘Spectra’ or ‘Properties’ views of the ‘Visualize’ page brings up the detailed information about the sample library from the Filter page. Similarly, clicking on the ‘Library Summary’ button leads to a summary page with the sample library information, properties, and spectra. For example, Fig. 6 shows part of the library summary view for one Zn-Ni-Co-O sample library (https://htem.nrel.gov/#/samples/6701). According to XRF results (Fig. 6a), there is a linear Zn composition gradient across the sample library (color scale), whereas the thickness does not change much across the sample library (point size). The conductivity (Fig. 6b) also changes across the sample library (color scale), whereas the direct band gap remains approximately constant (point size). Putting together these two datasets (Fig. 6c), it becomes clear that the conductivity (plotted on the logarithmic scale) reaches the maximum value of 4 S/cm at the Zn composition of 33%, regardless of direct band gap (color scale) and the thickness (point size). The underlying XRD patterns (Fig. 6d) and optical absorption spectra (Fig. 6e) can also be displayed. This example illustrates how the ‘Visualize’ page can be used to explore and analyze the data in HTEM DB. More types of properties and spectra will be added to the database in the future.

Since the HTEM DB web interface (https://htem.nrel.gov/) is meant for basic exploration of data, its data analysis functionality is limited to making simple property and spectra plots within a few datasets, as described above. For more detailed analysis beyond what’s available in the HTEM DB web interface, the users can download the datasets of interest (Data Citation 1), and analyze them using the software packages of their choice (MatLab, Mathematica, Igor, R, Python etc). This can be accomplished using ‘Download’ buttons on the property and spectra pages (also planned for filter page), that save the data in simple CSV format. For easier analysis across larger number of datasets, users are encouraged to use a full featured and open API (https://htem-api.nrel.gov/), where data is also available in several other commonly used materials-specific formats, such as NIST’s MDCS format^26^ and Citrine’s MIF format^27^. The API data access rather than data download is recommended for the most current results, since HTEM DB content is updated regularly, as additional data becomes available, as data processing bugs are fixed, and as data is curated based on its consistency.

### Data Mining and Machine Learning

The HTEM DB provides researchers an opportunity to explore composition, structure and properties of materials from a large number of diverse chemical systems, calling for application of data mining and machine learning techniques. In this section we discuss two examples of applications of such statistical methods to HTEM DB: (1) unsupervised learning (clustering) to support visualization and building understanding of the contents of the database (2) supervised learning (prediction) to build predictive models for key materials properties.

**Figure 7:**
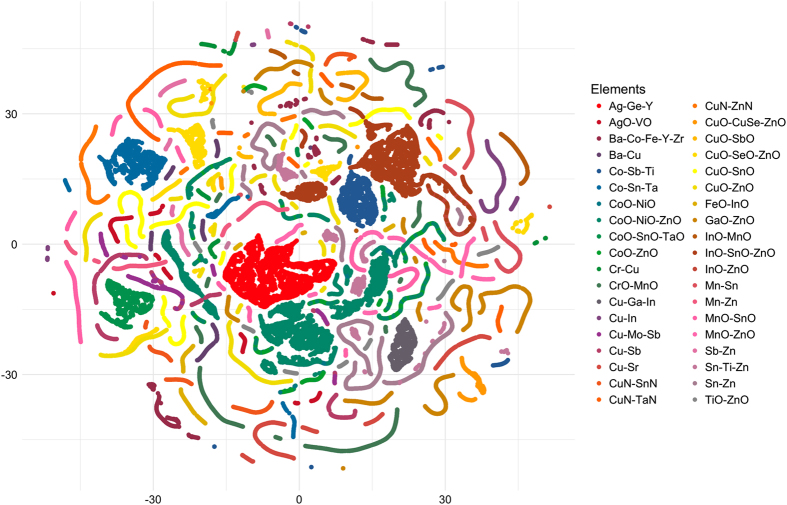
Visualization of most common compositions in the database (those with greater than 440 individual measurements) using the t-SNE dimensionality reduction algorithm. This visualization shows binary compounds as lines of points and ternary compounds as clouds of points. Using this technique, the complexities of the compositional space can be interactively visualized and explored in a single map.

One challenge that arises when analyzing large and diverse experimental datasets like the HTEM DB is inferring relationships between samples, and meaningfully grouping these samples. In practice, the database collects measurements from many isolated experiments in different chemical systems and with diverse research aims. In order to extract broad knowledge from the resulting collection of data, it is useful to group similar samples synthesized at different times and for different purposes. As shown in Fig. 7, we have attempted to visualize 70,000+ measured samples’ compositions in a single plot. The t-distributed stochastic neighbor embedding (t-SNE) algorithm with package-default settings collapses a sparsely populated 30+ dimensional compositional space into two dimensions, which may be visualized easily^28^. This approach is an application of unsupervised machine learning and dimensionality reduction, that has not been used before for machine learning in materials science^14^.

In the example shown in Fig. 7, the dominant compositional configuration is used to color points. As a result, individual materials are grouped so that their similarity (or difference) in the larger compositional space is still retained in this 2D visualization. Lines indicate (pseudo)binary composition systems (e.g., CoO-NiO, ZnO-CoO) while clusters of points indicate (pseudo)ternary compositions (e.g., ZnO-NiO-CoO). As such, this unsupervised machine learning algorithm simplifies the task of describing the database compositional contents in a single visualization, which allows a scientist to quickly determine if the chemical systems of interest is present in the database, and how it is related to the other database contents. For example, such visualization can help material scientists contributing to the database to understand what related areas have already been explored. As another example, data scientists may similarly use this visualization to better understand the coverage of the dataset, and how it may overlap or diverge from other available data.

Another important goal of materials science is to predict property values of materials that have not been measured yet. Whereas this can be done from first principles for certain materials properties (e.g., effective masses, band gaps), other properties may be too difficult or even impossible to compute (e.g., electrical conductivities, work functions). Even for some well-established theoretical property calculations (e.g., band gap), the computations are too expensive for certain classes of materials (e.g., non-stoichiometric compounds, alloys, compounds with open d- or f-electron shells) that constitute a substantial fraction of crystallographic databases. For all these cases, a possible alternative approach is developing predictive statistical models trained on previously measured experimental data to guide the search for new materials with desired properties. Such models are able to make thousands of predictions very quickly, which can provide insight into how the desired properties might vary across a broad materials landscape, and hence help honing experimental efforts to save resources. Open questions remain about the necessary size and diversity of training data to allow predictions of adequate fidelity. The large size of the underlying search space (number of potential materials) and the diversity/heterogeneity of the available training data present unique challenges in materials science.

**Figure 8:**
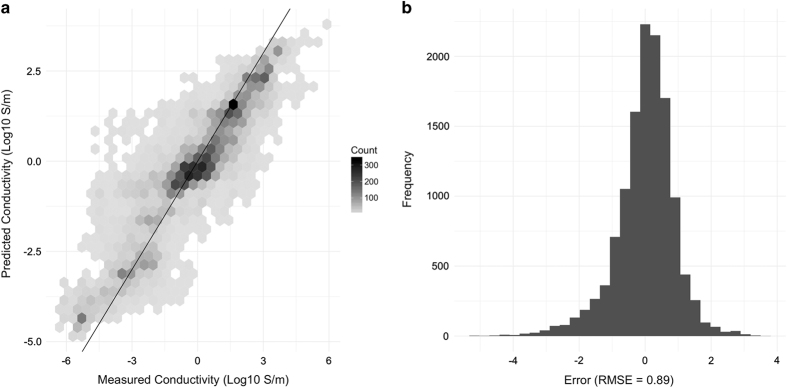
Performance of a Random Forest model for predicting material conductivity using measured composition. (**a**) Hex-binned scatterplot showing predictions compared to 16,093 measured values using the final model from sample-based cross validation scheme. (**b**) Histogram of the same, showing an aggregate RMSE of 0.89 (log scaled) across the entire data. This performance should be considered as ‘best case’ compared to the performance from other training datasets and other cross-validation schemes given in Table 1.

One property of particular interest for electronic and energy applications that is included in the HTEM DB is electrical conductivity (inverse of thickness-normalized sheet resistance), measured in Siemens per centimeter (S/cm). This is an exponentially varying quantity with values ranging from 10^7^ S/cm for a highly conductive material, to 10^−3^ S/cm for a semiconductor material, to 10^13^ S/cm for a pure insulator. Figure 8 shows the conductivity predicted using random forest model^29^ based on the composition features using samples based cross-validation, compared to the measured conductivity for entries in the database with the entire training set. While these initial results are promising, allowing conductivity prediction within 1-2 orders of magnitude (Fig. 8b), some risk of overfitting compared to other cross-validation schemes exists. In the future, we plan to improve the accuracy and reduce overfitting. We would also like to utilize these machine learning models to create much larger ‘approximate’ databases with predicted properties for all composition and structure entries in the ‘exact’ database, in order to support data mining and knowledge discovery.

## Discussion

In this paper, we report on our progress towards the High Throughput Experimental Materials Database (HTEM DB). The HTEM DB is a large open experimental materials data resource that currently contains >140,000 entries, for which synthesis conditions, chemical compositions, crystal structures, and optoelectronic properties (e.g. electrical conductivity, band gaps) are reported; more than a half of property data is publicly available. As such HTEM DB aims to fill the gap between existing experimental structure databases (ICSD, ICDD) and computational property databases (Materials Project, Aflowlib) in the future. In addition, HTEM DB is expected to play a role in emerging materials ‘virtual laboratories’ or ‘collaboratories’^30^, and empower the reuse of the high-throughput experimental materials data by researchers that did not generate it. This includes both data scientists without materials science domain knowledge, and materials scientists without access to expensive high-through synthesis and characterization equipment.

The HTEM DB can be accessed via an interactive web application at htem.nrel.gov. This web interface allows searching by elements, filtering of sample libraries, visualization of properties and spectra, as well as download of the data, as illustrated here on the example of a Zn-Ni-Co-O dataset. As an example of data mining application of HTEM DB through application programming interface (htem-api.nrel.gov) we show clustering of ~70,000 entries using unsupervised t-SNE algorithm for data dimensionality reduction. We also demonstrate prediction of electrical conductivity with a root mean square error of 1 order of magnitude for a material property that varies over 8 orders of magnitude in the training dataset.

We attempted to make HTEM DB concurrent with the recently postulated FAIR data principle^31^, which calls to make scientific data Findable, Accessible, Interoperable, and Reusable. To make the data findable, each sample library in HTEM DB is assigned a unique number (e.g., 6701 for the sample library presented here), that can be linked to a persistent identifier (e.g., DOI or handle). To ensure that the HTEM DB data is accessible, it can be retrieved through either web user interface (web-UI, https://htem.nrel.gov) or through application programming interface (API, https://htem-api.nrel.gov/). The interoperability of the data is ensured through the download of files with unique sample identifiers in materials-specific XML-based and JSON-based formats through the API (Data Citation 1). To enable the reuse of the HTEM DB data, the materials property data are described with many metadata attributes such as deposition conditions, and the associated dates, names, instruments. The application of supervised and unsupervised machine learning algorithms presented in this paper are examples of the HTEM DB data reuse.

The HTEM DB builds on nearly a decade of systematic data management and high-throughput experiments at NREL. In the methods section, we describe this enabling materials data infrastructure^32^, including the laboratory information management system (LIMS) and the resulting HTEM database architecture, as well as high-throughput experimentation techniques used to generate data in HTEM DB. However, it is important to note that HTEM DB would not have been possible to build without dedicated seed investment into this materials data infrastructure effort. Now that the HTEM DB infrastructure exists, only limited investment is needed to add more data to it, as suggested by the Data Management plans of the agencies that fund materials research and development. This emphasizes the importance to seed and sustain funding to materials data infrastructure, in addition to mandating that materials data is made publicly available. There is no doubt that materials data is a strategic asset for the materials science community, so appropriate resources should be allocated support to its management, curation, and preservation.

## Method

### High-Throughput Experimental Methods

As shown in Fig. 2, the PVD synthesis methods used to prepare the sample libraries described in HTEM DB include co-sputtering and pulsed laser deposition (PLD), both performed in vacuum chambers. In both of these PVD methods, solid material precursors (often metallic or ceramic disks referred to as ‘targets’) are converted in vacuum into plasma phase by external energy stimuli (electrical radio-frequency power in sputtering, and pulsed laser power in PLD). The resulting vapor condenses on a smooth support (often square or round plates of glass or silicon, referred to as ‘substrate’), forming a thin film coating on its surfaces. In high-throughput or ‘combinatorial’ sputtering methods, the intentional changes in composition across the coating are obtained by placing the targets at an angle with respect to the substrate; in combinatorial PLD, a similar effect is achieved by repetitive synchronized switching of the targets and rotation of the substrate. The ease of formation of such composition gradients in these PVD methods is an important advantage for high throughput experimentation. Another advantage is that sputtering is a common industrial technique for deposition of coatings, so the material discoveries made in the lab can be translated to manufacturing scale.

The spatially-resolved characterization techniques used to characterize the sample libraries in HTEM DB currently include x-ray fluorescence (XRF), x-ray diffraction (XRD), electrical (ELE) and optical (OPT) measurements. The XRF and XRD use x-rays to measure chemical composition and thickness of the thin film samples, as well as to identify their crystal structure and access their degree of crystallinity. This information is needed to understand how the microstructure and other imperfections of the synthesized samples differ from the idealized material defined by its atomic constituents and their spatial arrangement. The electrical four-point probe instrument supplies current and measures voltage to determine the sheet resistance of the samples, and the optical spectroscopy uses ultraviolet, visible and infrared light to measure transmittance and reflectance spectra of the samples. From these two optical and electrical measurements, the absorption spectra (including the band gap) and the electrical conductivity/resistivity are calculated, using thickness of the samples determined by XRF. These measurements supply insight into the optoelectrical properties, which are essential for determining which material may be best suited to a given application.

The term ‘high-throughput’ in the HTEM DB name refers to the accelerated rate of experimentation within the material system of choice, as defined by the combination of chemical elements, using a gradient to synthesize a large number of materials in a single step. The choice of the material system has lower throughput, and has typically been based on theoretical calculations or prior experimental literature, and on the likelihood to have the desired properties. In turn, the desired properties are determined by addressing the specific technological needs of the funding agencies that support the work. For example, Co-based oxide materials systems in HTEM DB (Co-O, Co-Zn-O, Co-Ni-O, Co-Zn-Ni-O) have been predicted by first-principles calculations^33^, and studied as p-type transparent conductors for electrical contact applications in solar cells^34^. Thus, the HTEM DB is a collection of data from multiple funded research projects on different materials systems of choice, rather than an effort to systematically cover all possible combinations of element in the periodic table.

It is also worth noting that that high-throughput experiments accelerate exploration of only the variables that are amenable to high-throughput experimentation, such as chemical composition or substrate temperature. Tuning other processing parameters can, and often does, lead to further improvement in the materials properties. Taking the aforementioned Zn-Ni-Co-O dataset as an example, the broad survey of compositions shows that the highest electrical conductivity of 70 S/cm occurs at Ni/Co-rich compositions^35^. However, further optimization of the thin film deposition process and introducing post-deposition annealing can increase the conductivity to 170 S/cm^36^. This illustrates that HTEM is meant for exploration of broad trends and correlations across the data, to identify the most promising regions of chemical compositions and processing parameters for further optimization.

### Database architecture and information management system

As shown in Fig. 4, HTEM DB is built upon NREL’s laboratory information management system (LIMS) for materials science, which is also used for regular (non-HTE) materials science data. In this system, each synthesis and characterization instrument is attached to a control computer that is responsible for capturing the data. An automatic harvesting system located on a central server and implemented in C++ collects data into a central repository, parses the file formats, and indexes the data in a data warehouse for archiving^37^. The warehouse enables researchers to access their data remotely without the need to go to each individual computer that are used to control the synthesis and characterization instruments. NREL researchers can access the data warehouse through an internal web site that organizes the files hierarchically by lab, instrument, and time. Users can also search for files by name, date ranges, or by metadata. The site also provides the ability to track samples and projects allowing users to associate files with the target samples. Visualization of the data files has been built into the site for some instruments.

For this particular project, we leveraged this LIMS system to construct a specialized database for high-throughput experimental materials (HTEM) results using standard object-relational database architecture. This involved an extract-transform-load (ETL) process of curating and aligning existing characterization data as well as building new systems for capturing deposition metadata. The ETL process is implemented using a combination of C, Python, and Ruby and works in three stages. The first stage searches for any sample data matching a set of file naming patterns within the data warehouse. The second stage processes and reformats the data using a toolkit of custom-built software that is able to extract data from numerous formats, some proprietary. Finally, in the third stage, the aligned data is cleaned and coalesced, so that the most recent data is stored in the database for easy access. To aid efficient metadata collection for stage-1, Graphical User Interfaces (GUIs) at the computers attached to the synthesis and characterization instruments were developed. In addition, this GUI found essential for collecting the material ‘synthesis recipes’ that are needed for data mining and analysis.

The web-based user interface to the HTEM database provides a way for both internal and external researchers to discover, search and analyze the final data. This application is custom-built using NodeJS and the Aurelia front-end framework to provide a highly interactive, modern interface. Users can select sample libraries based on a number of filters and plot their properties and measured spectra side by side. The property and spectra plotting features are implemented using Google Chart tools. Those who want to explore the data further, can download it directly (Data Citation 1) or access it programmatically through the API at htem-api.nrel.gov. Data access as opposed to data download is recommended to ensure it is up to date and accurate, since HTEM DB is being updated on the regular basis. The same application programming interface (API) allows consistent interaction between client applications (e.g., analysis programs, the web application and visualizations) and the HTEM database.

### Machine Learning Methods

We used a random forest supervised learning technique to build a model for electrical conductivities in HTEM DB, and evaluated its accuracy in making predictions through repeated random subsample cross validation where a random selection of 25% of data points (without replacement) are withheld from training for testing. This procedure is repeated 10 times to ascertain the average accuracy of the method. Fitting was performed using the R language with randomForest and caret packages with default starting parameters^38^. Because there is substantial similarity of the samples on a given sample library, we must be careful to avoid overfitting the data. To address this concern, in cross validation, we perform three kinds of withholding from least to most conservative: 25% of random sample entries (risks overfitting), 25% of samples libraries, 25% of published studies (groups of sample libraries, risks bias). Table 1 provides accuracy for different training sets.

In alignment with the different expected usage scenarios, we trained the model separately on three sets of features: (1) sample deposition information including temperature, pressure, time, sputtering compounds and gasses present, (2) experimentally measured composition describing the fractional elemental composition obtained through x-ray fluorescence combined with elemental descriptors^39^, (3) structural features including x-ray diffractogram peak locations, intensities, etc. To ascertain ‘best case’ performance, we also trained the model on all feature sets combined. Combining the three models, a researcher might search within a structural and compositional space to determine a range of promising synthesis approaches to achieve a particular property (in this case, conductivity). Being able to predict conductivity from any of these data would allow researchers to estimate conductivity of the deposited materials for which some composition/structure measurements have been made, while conductivity has not been measured.

## Additional information

**How to cite this article**: Zakutayev, A, et al. An open experimental database for exploring inorganic materials. *Sci. Data* 5:180053 doi: 10.1038/sdata.2018.53 (2018).

**Publisher**’**s note**: Springer Nature remains neutral with regard to jurisdictional claims in published maps and institutional affiliations.

## Figures and Tables

**Table 1:** Performance of a random forest model predicting conductivity given different feature-sets and cross validation resampling schemes.

**Feature Set**		**Compositional (RMSE/SD,mtry)**	**Structural (RMSE/SD, mtry)**	**Synthesis (RMSE/SD, mtry)**	**Combined (RMSE/SD, mtry)**
Cross Validation Method	Positions (samples)	1.35/0.02, 2	2.15/0.01, 16	0.976/0.03, 17	0.67/0.03, 129
	Sample Libraries	1.68/0.13, 2	2.25/0.12, 2	1.93/0.11, 2	1.61/0.15, 2
	Studies	1.83/0.48, 2	1.94/0.42, 2	1.93/0.55, 2	2.05/0.55, 2
The root mean square error (RMSE) and standard deviation (SD) provides the average error and variance in log10(S/m) for the cross validation. A parameter sweep was used to determine the best value for mtry, which is the number of randomly chosen parameters at each step of the tree building algorithm. The models tuned using per-position cross validation tend to overfit the data with a larger mtry, while mtry=2 performs well for the more conservative resampling schemes.					
